# Antibodies against Food Antigens in Patients with Autistic Spectrum Disorders

**DOI:** 10.1155/2013/729349

**Published:** 2013-08-01

**Authors:** Laura de Magistris, Annarita Picardi, Dario Siniscalco, Maria Pia Riccio, Anna Sapone, Rita Cariello, Salvatore Abbadessa, Nicola Medici, Karen M. Lammers, Chiara Schiraldi, Patrizia Iardino, Rosa Marotta, Carlo Tolone, Alessio Fasano, Antonio Pascotto, Carmela Bravaccio

**Affiliations:** ^1^CIRANAD, Second University of Naples, via Pansini 5, Building 3, 80131 Naples, Italy; ^2^“Alimenti e Salute” PhD School, CIRANAD, Second University of Naples, via Pansini 5, Building 3, 80131 Naples, Italy; ^3^Department of Experimental Medicine, Second University of Naples and Centre for Autism, La Forza del Silenzio, via S. Maria di Costantinopoli 16, 80138 Naples, Italy; ^4^Department of Mental and Physical Health and Preventive Medicine, Second University of Naples, Largo Madonna delle Grazie 1, 80138 Naples, Italy; ^5^Department of General Pathology, Second University of Naples, Larghetto S. Aniello a Caponapoli 2, 80138 Naples, Italy; ^6^Center for Celiac Research and Mucosal Immunology and Biology Research Center, Massachusetts General Hospital East, Charlestown, MA 02129-4404, USA; ^7^Department of Experimental Medicine, Second University of Naples, via S. Maria di Costantinopoli 16, 80138 Naples, Italy; ^8^UOC Clinical and Molecular Pathology, Second University of Naples, via S. Maria di Costantinopoli 16, 80138 Naples, Italy; ^9^Department of Psychiatry, University of Catanzaro “Magna Graecia”, via T. Campanella, 88100 Catanzaro, Italy; ^10^Department of Woman, Children and of General and Specialistic Surgery, Second University of Naples, via L. De Crecchio 4, 80138 Naples, Italy; ^11^Department of Medical Translational Science, University of Naples “ Federico II ”, via Pansini 5, 80131 Naples, Italy

## Abstract

*Purpose*. Immune system of some autistic patients could be abnormally triggered by gluten/casein assumption. The prevalence of antibodies to gliadin and milk proteins in autistic children with paired/impaired intestinal permeability and under dietary regimen either regular or restricted is reported. *Methods*. 162 ASDs and 44 healthy children were investigated for intestinal permeability, tissue-transglutaminase (tTG), anti-endomysium antibodies (EMA)-IgA, and total mucosal IgA to exclude celiac disease; HLA-DQ2/-DQ8 haplotypes; total systemic antibodies (IgA, IgG, and IgE); specific systemic antibodies: **α**-gliadin (AGA-IgA and IgG), deamidated–gliadin-peptide (DGP-IgA and IgG), total specific gliadin IgG (all fractions: **α**, **β**, **γ**, and **ω**), **β**-lactoglobulin IgG, **α**-lactalbumin IgG, casein IgG; and milk IgE, casein IgE, gluten IgE, -lactoglobulin IgE, and **α**-lactalbumin IgE. *Results*. AGA-IgG and DPG-IgG titers resulted to be higher in ASDs compared to controls and are only partially influenced by diet regimen. Casein IgG titers resulted to be more frequently and significantly higher in ASDs than in controls. Intestinal permeability was increased in 25.6% of ASDs compared to 2.3% of healthy children. Systemic antibodies production was not influenced by paired/impaired intestinal permeability. *Conclusions*. Immune system of a subgroup of ASDs is triggered by gluten and casein; this could be related either to AGA, DPG, and Casein IgG elevated production or to impaired intestinal barrier function.

## 1. Introduction

Autism and autism spectrum disorders (ASDs) are complex neurodevelopmental conditions [1–4]. The aetiology and pathogenesis of ASDs are still poorly understood and the actually available therapeutical interventions are of behavioural, developmental and educational impact; no biomedical effective therapies are currently available.

It has been suggested that increased permeability of the gut and blood-brain barrier might be involved in the pathogenesis of these conditions [5]. Several studies suggest that autistic children could have an impaired gut barrier function as testified by an elevated intestinal permeability [5, 6]; the question if the intestinal barrier is impaired in ASD remains a debated question [7]. The impaired intestinal barrier function detected in ASD might predispose autistic children to sensitization to environmental antigens by allowing the passage of dietary-derived nonself antigens in the intestinal lamina propria, thereby triggering an immune response to these molecules [5, 6]. 

Recently, we have reported that intestinal permeability is increased in a large percentage of not-celiac autistic subjects and that it is partially corrected by gluten/casein-free diet regimen [8]. A 2009 review of clinical controlled studies on this topic showed that evidence for efficacy of these diets in ASDs is poor, and good quality randomized controlled trials are still needed [9]; afterwards, other studies were published partially confirming that some autistic children embracing a gluten-free diet show improvement in symptoms and social behaviours [10–12]. The fact that removing gluten from the diet may positively affect developmental outcome for some children with ASD suggests that autism may be part of the spectrum of not-celiac-gluten sensitivity (NCGS), at least in some cases. 

 Immunological alterations and changes in innate and adaptive immunity might play a role in the pathogenesis of autism [13, 14]. Although many studies described impaired response of immune system in ASD [13–22], reactions toward food antigens, such as gluten and casein involved in the previously mentioned diet regimen, have been poorly described. Lucarelli et al. [22] found high levels of IgA antigen specific antibodies for casein, *α*-lactalbumin, and *β*-lactoglobulin and IgG and IgM for casein. PBMCs obtained from ASD children produced more tumor necrosis factor-a (TNF-a)/interleukin-12 (IL-12) than those obtained from control subjects when challenged with casein, *β*-lactoglobulin, and *α*-lactalbumin [18]. Specific IgG to gluten was comparable among ASDs and controls [15]. A significant percentage of autistic sera were associated with elevated immunoglobulin IgG, IgM, or IgA antibodies against gliadin [21].

Keeping in mind that autism is caused by the interaction between genetic and environmental factors [1], all these findings might suggest that sensitivity related illness (SRI) [23] affects some autistic children; SRI initiates with a toxicant induced loss of tolerance (TILT) and sensitivity to gluten/casein could only be one of the triggering aspects. In this view removing the triggering agents from the diet could be considered as a tool in nutrition therapy for ASDs. 

To challenge the hypothesis that dietary-derived nonself antigens could permeate impaired gut barrier and stimulate immune system, we evaluated the antibody prevalence to a series of dietary proteins including gluten/gliadin and milk proteins in a group of autistic children with increased/normal intestinal permeability fed either a regular alimentary regimen or a gluten-casein free diet (GF/CF).

## 2. Methods

### 2.1. Subjects

 One hundred sixty-two consecutive subjects with ASDs (131 boys and 31 girls with a mean age of 7.4 ± 5.1 yr) were recruited from either the outpatient or inpatient services of the Child and Adolescent Neuropsychiatry Unit at the Second University of Naples, Italy. The cohort included 5 couples of twins. For all patients, diagnosis of ASD was made according to the Diagnostic and Statistical Manual of Mental Disorders, Fourth edition, Text Revision (DSM-IV-TR). The compliant enrolled children were administered the Autism Diagnostic Interview-Revised version, the Childhood Autism Rating Scales, and the Autism Diagnostic Observation Schedule-Generic to verify the diagnosis of autism [24–27]. A carefully detailed gastrointestinal (GI) anamnesis was obtained for each subject, with specific emphasis to type of special diet (e.g., GF/CF diet) and reported food intolerances. Among the enrolled ASDs, 31 embraced a gluten/casein free diet (GF/CF) for an average time of 3 years. The correct and effective implementation of the GF/CF diet was under the responsibility of the parents and assumed from their report; only those who reported ≤1 transgression/month and were on GF/CF since at least 1 year were enclosed in the GF/CG group. Among the enrolled ASDs, 100 children presented with GI symptoms, as reported during administration of a specific questionnaire to the parents. Altered bowel movements were reported as constipation (27,3%) more frequently than diarrhea (8,4%). Other symptoms (26,0%) included pain, flatulence, prevalence of alternating constipation/diarrhea, and gastric reflux. Exclusion criteria were autisms secondary to genetic syndromes; Rett syndrome; childhood disintegrative disorder; epilepsy; neurological syndromes; BMI < 25th or >85th percentile; concomitant condition of known celiac disease (CD) concomitant condition of major diseases of the intestinal tract such as inflammatory bowel disease or hepatic disorders, and known and serologically proven food intolerances. 

Forty-four healthy children (27 boys and 17 girls with a mean age of 7.1 ± 3.1 yr) were recruited as matched controls among families of doctors, nurses, students of the Child and Adolescent Neuropsychiatry Unit and Gastroenterology at the Second University of Naples, Italy. None of these healthy children claimed any chronic and/or recent GI symptoms and were not affected by any major GI disease. A carefully detailed GI anamnesis was obtained for each subject, with specific emphasis to type of special diet (e.g., GF/CF diet) and reported food intolerances. All of them were on regular diet (i.e., inclusive of gluten and milk proteins). The exclusion criteria were a concomitant condition of known celiac disease (CD), a concomitant condition of major diseases of the intestinal tract such as inflammatory bowel disease or hepatic disorders, and known and serologically proven food intolerances. 

Informed consent was obtained from the parents of all children before starting any procedure. The study was approved by the ethics committee of our department and was carried out in accordance with the Helsinki Declaration of 1975.

### 2.2. Investigated Parameters

#### 2.2.1. Anti-Tissue Transglutaminase (tTG)-IgA, Anti-Endomysium (EMA)-IgA, and Total IgA Antibodies

To rule out celiac disease (CD), tTG-IgA, EMA-IgA, and total IgA antibodies were determined. The quantitative determination of specific IgA antibodies against human recombinant tissue transglutaminase (tTG) in serum was achieved by means of a sandwich type enzyme immunoassay and a detection system in colorimetry (Eurospital, Italy). Normal values were set as <9 U/mL. The detection of class IgA anti-endomysium antibodies (EMA) was performed by indirect immunofluorescence on sections of human umbilical cord (Eurospital, Italy). The antigen-antibody complex was visualised by fluorescence microscope with the aid of fluorescein-labelled antibody. The detection of total IgA serum antibodies was achieved by immune-turbid metric test system (Roche Diagnostics Gmbh, Cobas 6000, Germany). IgA antibodies reacted with the antigen, forming an antigen-antibody complex. Agglutination was measured metrically. Age based normal ranges of total IgA are reported in Supplementary Material available online at http://dx.doi.org/10.1155/2013/729349.

#### 2.2.2. HLA Typing

HLA-DQ2 and -DQ8 loci were typed in all ASDs children. Genomic DNA was extracted and purified from blood samples collected in EDTA by means of a commercially available kit (Eugen-Estraction, Eurospital, Italy). HLA-DQ2 and -DQ8 loci were typed using commercial kits (EU-Gen Risk, Eurospital, Italy).

#### 2.2.3. Intestinal Permeability

Intestinal permeability was assessed with the lactulose/mannitol (LA/MA) test. The test was administered once to all recruited children. The LA/MA test is considered a valuable and noninvasive test for monitoring barrier function of the small intestine. The procedure is based on the simultaneous oral administration of 2 sugar probes of different molecular sizes and absorption routes and the assessment of the concentration of each molecule in the urine. The LA/MA test was administered as previously described [8]. Briefly, an oral isosmolar load of the 2 probes—5 g of lactulose (LA) and 2 g of mannitol (MA)—is orally administered to fasting subjects, and urine samples are collected for the following 5 h. The LA/MA detection in the urine samples was performed by high-performance anion exchange chromatography with pulsed amperometric detection, as previously described [28]. Intestinal permeability is expressed as the ratio of the recovered percentage of lactulose versus mannitol (LA/MA). The cut-off value for the normal range was set at LA/MA < 0.030 [8].

#### 2.2.4. Antibodies to Food Antigens

Antibodies against *α*-gliadin, anti-*α*-gliadin (AGA)-IgG and -IgA, and anti-deamidated *α*-gliadin peptides (DPG)-IgA and -IgG, were evaluated. The quantitative determination of specific IgA and IgG antibodies against *α*-fraction of wheat gliadin (AGA) in serum was achieved by means of a sandwich type enzyme immunoassay and a detection system in colorimetry (Eurospital, Italy). Normal values were set as <15 U/mL and <50 U/mL, respectively. The quantitative determination of IgA and IgG antibodies directed against the deamidated *α*-gliadin peptides (DPG) in serum was achieved by means of a sandwich type enzyme immunoassay and a detection system in colorimetry (Eurospital, Italy). Normal values were set as <5.5 U/mL and <10 U/mL, respectively.

To determine whether the children reacted to food allergens, total IgE and IgG were assessed. In addition, specific IgE (*β*-lactoglobulin IgE, *α*-lactalbumin IgE, gluten IgE, casein IgE, and milk IgE) and specific IgG (*β*-lactoglobulin IgG, *α*-lactalbumin IgG, gliadin IgG (all fractions: *α*, *β*, *γ*, and *ω*), and casein IgG) were detected (Phadia, Italy). The detection of total IgG serum antibodies was performed by immune-turbidimetric test system (Roche Diagnostics Gmbh, Cobas 6000, Germany). IgG antibodies reacted with the antigen forming an antigen-antibody complex. Agglutination was measured metrically. The total IgE and specific IgG and IgE serum antibodies to gliadin/gluten, casein, *α*-lactalbumin, and *β*-lactoglobulin were detected by FEIA method, an enzyme immunoassay system with fluorometric detection through the use of equipment ImmunoCAP (Phadia, Uppsala, Sweden). Normal values for specific IgG were set as <12 mgA/L. Age-based normal ranges of total IgG and IgE are reported in Supplementary Material.

#### 2.2.5. Statistical Analyses

Statistical significance was assessed at a level of *P* < 0.05. Variables were summarized either as mean ± SEM, mean ± SD, or percentage. The Mann-Whitney test or Kruskal-Wallis and Dunn's multiple comparison test were used to evaluate the differences amongst means. Fisher exact test was applied to compare group frequencies. Data handling and analysis were performed through Graph Pad Prism 5 (GraphPad Software Inc., La Jolla, CA, USA).

## 3. Results

### 3.1. CD Screening

Among the 162 recruited ASDs patients, two children resulted serological positive for CD (positive EMA IgA, tTG-IgA >9 U/mL) and were therefore excluded from the study. None of the 44 investigated healthy children tested positive to CD serology. Mean serum tTG-IgA values were 1.8 ± 1.5 U/mL and 2.1 ± 2.2 U/mL in ASDs and healthy children, respectively (mean ± SEM). Total IgA resulted decreased in 2 and increased in 13 ASDs (Table 2) with no difference versus controls. Forty percent of the ASDs children carried the HLA DQ2/DQ8 haplotype (Table 1).

### 3.2. Intestinal Permeability

**Figure 1:**
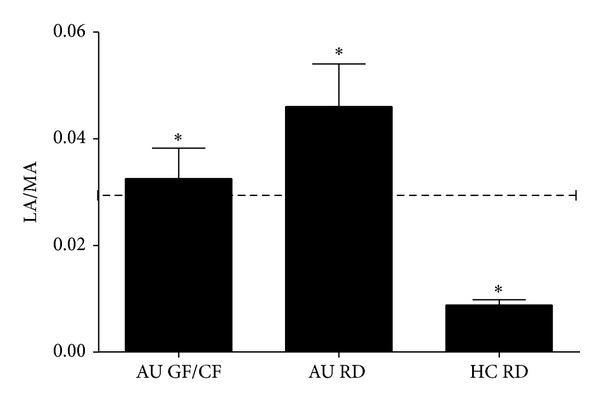
Intestinal permeability was evaluated by means of the LA/MA ratio [28] in ASDs children on a regular diet (AU RD) and on a GF/CF diet (AU GF/CF) and in healthy children all being on regular diet (HC RD). Normal range LA/MA cutoff is indicated by the dotted line. Values are given as mean ± SEM of the three groups. Significant differences were found between all groups (Kruskal Wallis-test and Dunn's multiple comparison test, *P < 0.0001).

 Intestinal permeability, evaluated by the LA/MA test, resulted different among the three groups (*P* < 0.0001 Kruskal-Wallis test with Dunn's Multiple Comparison Test): increased in ASDs children on a regular diet (0.046 ± 0.010) versus healthy children (0.009 ± 0.001) and ASDs on GF/CF diet (0.033 ± 0.006) (Figure 1). LA/MA test is considered altered when values >0,030 (cut-off value), as already assessed [8, 28]; 41 ASDs and 1 control had LA/MA values higher than the cutoff (Table 2). 

### 3.3. Anti-*α*-Gliadin (AGA) IgA and IgG

#### 3.3.1. Effect of GF/CF Diet

**Figure 2:**
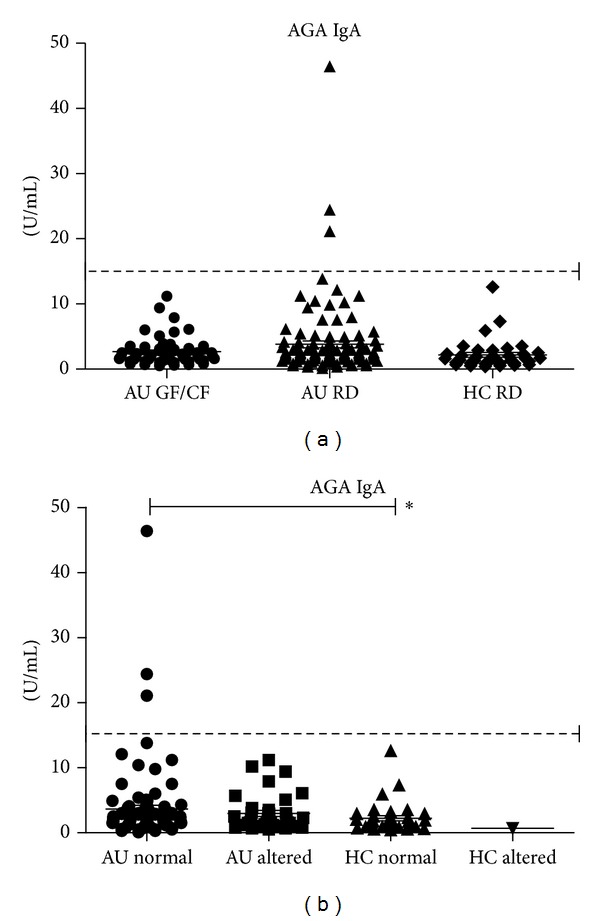
Mean ± SEM and individual titers of AGA-IgA (anti-*α*-gliadin IgA) are reported; normal range cutoff is indicated by the dotted line (15 U/mL). (a) The investigated subjects were divided in three groups to be compared on the basis of diet regimen: ASDs children on a regular diet (AU RD) or on GF/CF diet (AU GF/CF) and healthy children (HC RD); no significant differences were shown. (b) The investigated subjects were divided in four groups to be compared on the basis of normal/altered intestinal permeability: ASDs children with normal (AU normal) or altered (AU altered) LA/MA values and healthy children with normal (HC normal) or altered LA/MA values (HC altered); no significant differences were shown.

The prevalences of AGA-IgA in ASDs children, whether on a regular diet or on GF/CF, and that in healthy children were similar; it was below cutoff but for three cases among ASDs on RD (Figure 2(a)). 

**Figure 3:**
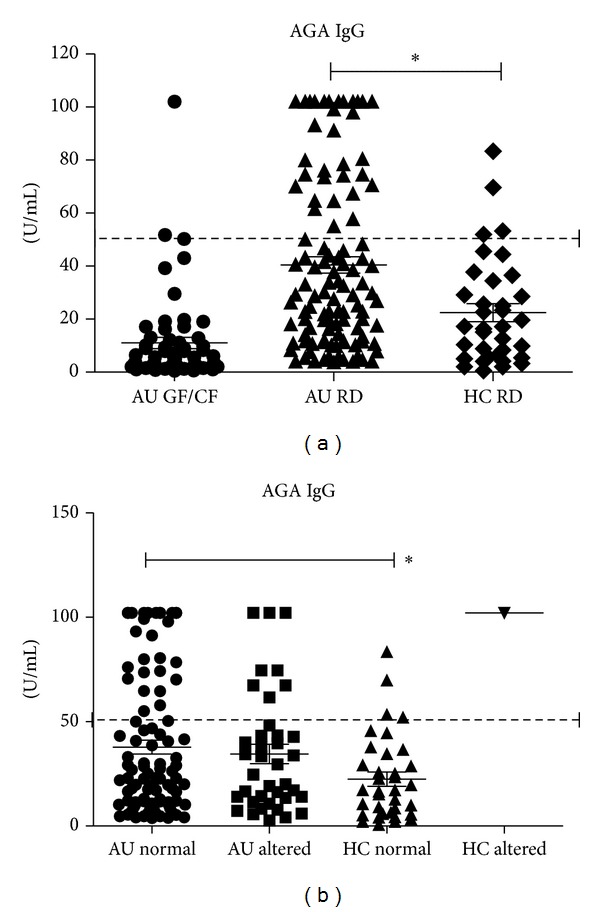
Mean ± SEM and individual titers of AGA-IgG (anti-*α*-gliadin IgG) are reported; normal range cutoff is indicated by the dotted line (50 U/mL). (a) The investigated subjects were divided in three groups to be compared on the basis of diet regimen: ASDs children on a regular diet (AU RD) or on GF/CF diet (AU GF/CF) and healthy children (HC RD); AGA-IgG were increased in ASDs versus healthy children (*P < 0.005, Kruskal Wallis test, Dunn's multiple comparison test). (b) The investigated subjects were divided in four groups to be compared on the basis of normal/altered intestinal permeability: ASDs children with normal (AU normal) or altered (AU altered) LA/MA values and healthy children with normal (HC normal) or altered LA/MA values (HC altered); AGA IgG titers in ASDs were increased irrespective of intestinal permeability (*P = 0.0018, Mann Whitney test).

AGA-IgG resulted more frequently increased in ASDs children on regular diet compared to healthy children (Table 2, *P* = 0.0203, Fisher's exact test). Mean values also were significantly different (40.4 ± 3.1 U/mL versus 22.4 ± 3.4 U/mL, *P* < 0.005), (Figure 3(a)). AGA-IgG mean titer decreased in ASDs children on a GF/CF diet (11.1 ± 3.2 U/mL); however, few ASDs still showed titers above normal values (Figure 3, Table 2). 

#### 3.3.2. Influence of Intestinal Permeability

The prevalence of AGA-IgA in ASDs children, whether they had a normal or increased intestinal permeability, and that in healthy children, was similar and below cut-off level (Figure 2(b)) but for the three above mentioned cases. AGA-IgG were increased in ASDs children with normal intestinal permeability as compared to healthy children with normal intestinal permeability (37.8 ± 3.3 U·mL versus 22.2 ±3.1 U/mL, *P* = 0.0018). The AGA-IgG titer in ASDs children with a normal intestinal permeability was similar to that in ASDs children with an increased intestinal permeability (33.1 ± 4.5 U/mL) (Figure 3(b)). 

Taken together, these results indicate that AGA-IgG titers are higher in ASDs compared to controls and are not influenced by changes in intestinal permeability; they are, however, partially influenced by diet regimen.

### 3.4. Anti-Deamidated *α*-Gliadin Peptides (DPG)-IgA and -IgG

#### 3.4.1. Effect of GF/CF Diet

**Figure 4:**
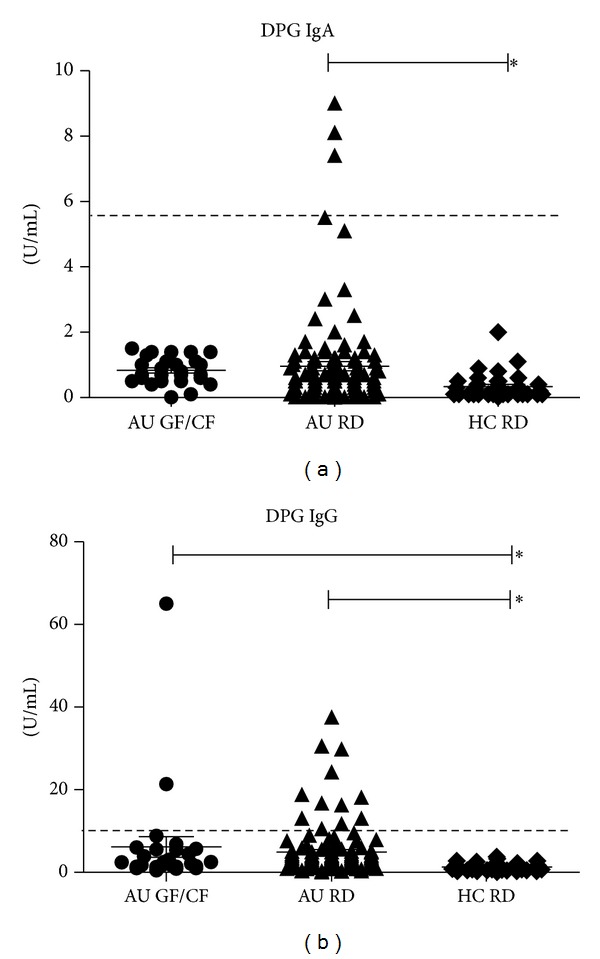
Mean ± SEM and individual titers of DPG-IgA and IgG (anti-deamidated gliadin peptides IgA and IgG) are reported. The investigated subjects were divided in three groups to be compared on the basis of diet regimen: ASDs children on a regular diet (AU RD) or on GF/CF diet (AU GF/CF) and healthy children (HC RD). (a) DPG-IgA values are reported; normal range cutoff is indicated by the dotted line (5,5 U/mL): significant differences were shown between AU RD and HC RD (*P < 0.0001 Kruskal Wallis test, Dunn's multiple comparison test). (b) DPG-IgG values are reported; normal range cutoff is indicated by the dotted line (10 U/mL): DPG-IgG were increased in ASDs versus healthy children (*P < 0.0001, Kruskal Wallis test, Dunn's multiple comparison test). Diet restrictions were ineffective in a few subjects.

DPG-IgA titers among ASDs on RD showed mean values significantly different than controls (AU RD: 0.960 ± 0.14; HC RD: 0.34  ± 0.07 U/mL; *P* < 0.0001) (Figure 4(a)). 

Among ASDs children, DPG-IgG mean values were increased compared to controls and only partially corrected by diet restrictions (Figure 4(b)). DPG-IgG mean values in both ASDs groups were 4.87 ± 0.6 U/mL (regular diet) and 6.14 ± 2.50 U/mL (GF/CF diet) and were significantly different from mean values in healthy children (1.27 ± 0.16 U/mL, *P* < 0.0001). DPG-IgG were more frequently increased in AU RD in respect to HC RD (Table 2, *P* = 0.0383, Fisher's exact test).

#### 3.4.2. Influence of Intestinal Permeability

The prevalences of DPG-IgA in ASDs children, whether they had a normal or increased intestinal permeability, and that in healthy children were similar. DPG-IgG mean values were similar in ASDs children with normal and increased intestinal permeability (4.79 ± 0.64 and 6.14 ± 1.94 U/mL) and in healthy children with normal intestinal permeability (1.27 ± 0.16 U/mL, *P* = NS). 

Taken together, these results indicate that DPG titers are higher in ASDs compared to controls and are not influenced by changes in intestinal permeability. 

### 3.5. Total and Specific IgG

 Total IgG resulted in the normal range (age based) in all subjects—ASDs and controls (Table 2).

**Figure 5:**
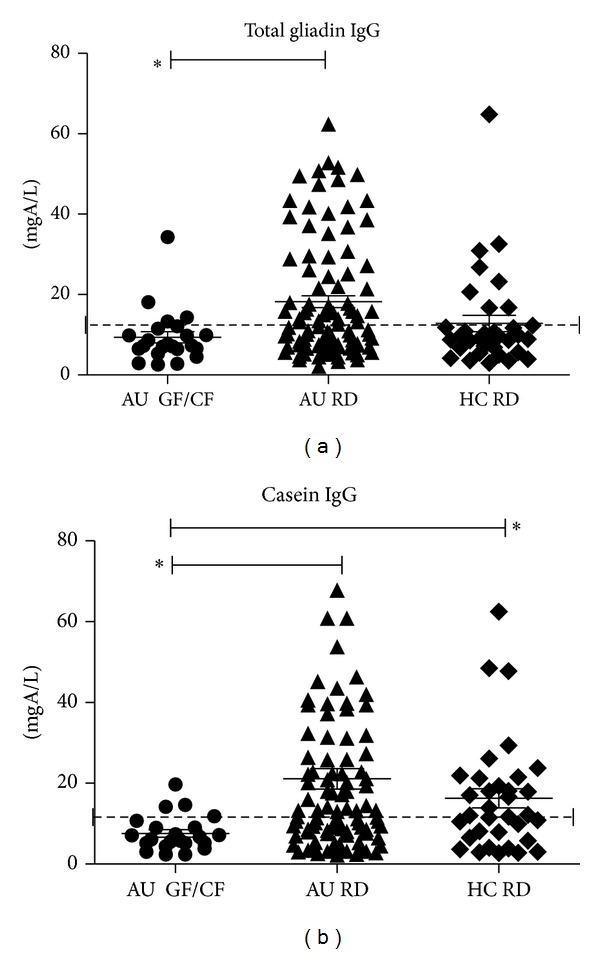
Mean ± SEM and individual titers of specific IgG are reported; normal range cutoff is indicated by the dotted line (12 mgA/L). The investigated subjects were divided in three groups to be compared on the basis of diet regimen: ASDs children on a regular diet (AU RD) or on GF/CF diet (AU GF/CF) and healthy children (HC RD). (a) Gliadin IgG were higher in ASDs on regular diet in respect to ASDs on GF/CF (*P < 0.0015 Kruskal Wallis test, Dunn's multiple comparison test). (b) Casein-IgG were lower in ASDs on GF/CF versus ASDs on regular diet and healthy children (*P < 0.0015, Kruskal Wallis test, Dunn's multiple comparison test).

 Among ASDs on RD, total gliadin IgG titers resulted >12 mgA/L more frequently and significantly than among controls (Table 2, *P* = 0.0043 Fisher's exact test), even if mean values were not significantly different (Figure 5(a); Table 2); GF/CF diet, as expected, influences Gliadin-IgG production (*P* < 0.007). 

Among ASDs on RD, casein IgG titers resulted >12 mgA/L more frequently and significantly than among controls (Table 2, *P* = 0.0034 Fisher's exact test), even if mean values are not significantly different (21.1 ± 2.5 and 16.3 ± 2.4 mgA/I, *P* = NS). The implementation of a GF/CF diet drastically and significantly decreased casein IgG titers (7.6 ± 0.9 mgA/I, *P* < 0.0015) (Figure 5(b)). 

Specific IgG titers to *β*-Lactoglobulin were similar between ASDs on RD and healthy children (AU RD: 19.0 ± 2.8 and HC RD: 15.0 ± 2.9). Specific IgG titers to *α*-lactalbumin were also similar between ASDs on RD and healthy children (AU RD: 19.2 ± 3.1 and HC RD: 14.3 ± 4.0). In both cases, diet restriction, as expected, drastically decreased IgG titers (Table 2).

#### 3.5.1. Influence of Intestinal Permeability

The prevalences of Casein-IgG in ASDs children, whether with normal or increased intestinal permeability (18.2 ± 2.7 and 19.4 ± 3.1 mgA/I), and that in healthy children were similar (15.5 ± 2.7 mgA/I, *P* = NS). Intestinal permeability alteration did not influence *β*-Lactoglobulin and *α*-Lactalbumin as well as total gliadin IgG titers (data not shown). 

### 3.6. Total and Specific IgE

#### 3.6.1. Effect of GF/CF Diet

**Figure 6:**
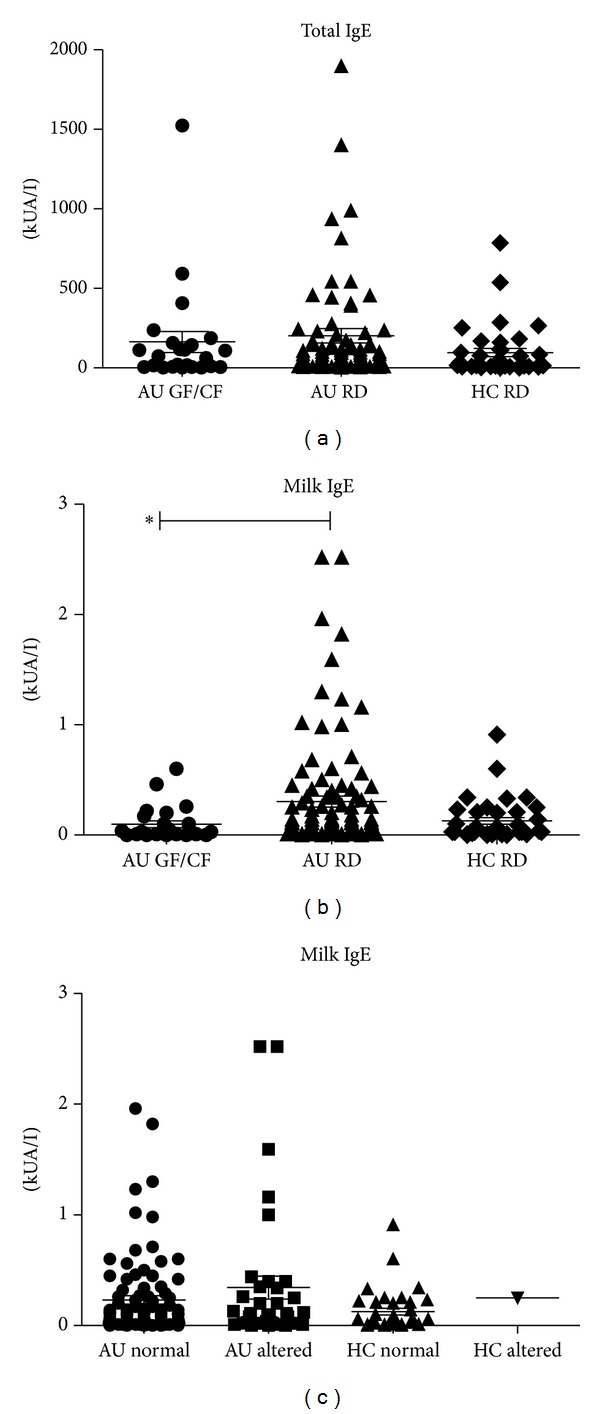
Mean ± SEM and individual titers of total IgE (a) and specific milk-IgE (b, c) are shown. The investigated subjects were divided in three groups (a, b) to be compared on the basis of diet regimen: ASDs children on a regular diet (AU RD) or on GF/CF diet (AU GF/CF) and healthy children (HC RD). The investigated subjects were divided in four groups (c) to be compared on the basis of normal/altered intestinal permeability: ASDs children with normal (AU normal) or altered (AU altered) LA/MA values and healthy children with normal (HC normal) or altered LA/MA values (HC altered). Specific milk-IgE titers in ASDs children on a regular diet resulted higher than ASDs on GF/CF (*P < 0.005, Kruskal Wallis test, Dunn's multiple comparison test).

Total IgE titers of ASDs children on a regular diet were similar to those of healthy children (202.5 ± 45.7 kU/I and 96.4 ± 26.4 kU/I, resp.) and were not influenced by a GF/CF diet (164.1 ± 65.8 kU/I, *P* = NS) (Figure 6(a)). Specific IgE to milk (0.30 ± 0.05 kUA/L) mean values were similar in ASDs on RD and healthy children (0.1 ± 0.0 kUA/I). They were reduced in ASDs children on a GF/CF diet (0.13 ± 0.03 kUA/L, *P* < 0.005) (Figure 6(b)).

#### 3.6.2. Influence of Intestinal Permeability

Total IgE and specific IgE milk in ASDs children were similar irrespective of normal (milk IgE: 0.23 ± 0.04 kUA/I) or increased intestinal permeability (milk IgE: 0.36 ± 0.12 kUA/I) (Figure 6(c)).

None of the other investigated specific IgE to *α*-Lactalbumin (AU RD: 0.22 ± 0.04; AU GF/CF: 0.07 ± 0.02; HC RD: 0.07 ± 0.02 kUA/I;), *β*-Lactoglobulin (AU RD: 0.15 ± 0.03; AU GF/CF: 0.07 ± 0.04; HC RD: 0.07 ± 0.02), Casein (AU RD: 0.11 ± 0.02; AU GF/CF: 0.03 ± 0.01; HC RD: 0.12 ± 0.08) and gluten (AU RD: 0.11 ± 0.02; AU GF/CF: 0.03 ± 0.01; HC RD: 0.80 ± 0.75) resulted different amongst the groups (Table 2), with regard to both diet restriction and intestinal permeability alteration. 

## 4. Discussion

Aim of this study was to investigate if an impaired intestinal barrier function might allow the passage of dietary-derived non-self antigens in the intestinal lamina propria triggering an immune response toward these molecules. To reach our goal we studied both small intestinal barrier function and immune response—general and specific toward dietary-derived non-self antigens in a large number of ASDs subjects; immunoglobulin titers were related to the paired/impaired barrier condition and to the dietary regimen either regular or restricted (gluten casein free diet). 

The condition of intestinal barrier impairment in ASDs is a largely debated question that has produced conflicting reports [7]. It was present [8, 29] in children with no concomitant gastrointestinal (CD, IBD) and hepatic disorders—that are usually associated with increased gut permeability—reporting gastrointestinal symptoms [8, 29]. We confirmed that a large percentage (25.6%) of the enrolled ASDs have an impaired intestinal barrier function, as assessed by the LA/MA test; GF/CF diet regimen tends to normalize the barrier impairment. 

Among the recruited children, the implementation of GF/CF diet was a free choice of the parents; this behaviour is frequently observed—19,4% of the population under study—and it derives from the parents subjective belief/observation that their children behaviour and GI disturbance ameliorate. From a scientific point of view this, also, is a debated question; few appropriate studies have been done, and the question is still under debate [7, 9–12]. In this study the GF/CF condition (deprivation of gluten and casein) was used to compare that of a regular diet in which gut lumen, barrier and immune system get in touch with these dietary-derived non-self antigens. Specific casein and gliadin IgG elevated titers resulted more frequent among ASDs on regular diet than controls (Table 2, Figure 5). The other investigated milk-derived antigens (Table 2) show patterns comparable to healthy controls. Previous findings on increased reactivity toward milk proteins (Casein) are confirmed [18, 22].

A more marked difference was found toward gliadin/gluten: AGA-IgG and even DPG-IgG increased titers were more frequently present in ASDs than in controls, in a few cases even independently of diet regimen. DPG are considered accurate predictors of CD in early infancy and a sign of gluten related intestinal damage [30]. Their synthesis “in vivo” is an expression of the interaction between tissue transglutaminase and gliadin peptides, rendering them highly specific toward deamidated gliadin; they can also be an involvement sign of adaptive immunity. The present finding of DPG-IgG alterations in not-celiac ASDs is important evidence in favour of the hypothesis that a subgroup of ASDs patients are specially triggered by gluten, as it was already suggested [8]. Possible cross reactivity can be excluded because gliadin is a prolamine, and gliadin derived peptides are peculiarly rich in proline, which makes it very specific to immune system. Moreover, it was demonstrated the presence of only a slight reaction of anti-cerebellar peptide to gliadin and absence of binding of anti-myelin basic protein (MBP), anti-milk, anti-egg, and anti-soy [21].

 Immunological alterations and changes in innate and adaptive immunity have been reported in individuals with ASDs [13, 14]; however, a “direct cause-and-effect relationship between immune dysfunction and ASDs has yet to be proven,” and “the role of immune responses in the pathogenesis of gastrointestinal disorders in individuals with ASDs warrants additional investigation” [7]. 

 Among ASDs children, low total IgG (hypogammaglobulinemia), with IgG subclass deficiency and selective IgA deficiency (<7 mg/dL), and elevated IgE were reported [13]. More recently, it has been demonstrated that autistic children have low-normal IgA mean levels compared to healthy age-matched controls, whereas serum IgE, IgG, and total gliadin specific IgG mean levels were comparable between both groups [15]. We did not find any statistically significant differences in IgA, IgE, IgG high titers frequency among the investigated subjects in respect to controls though we found an augmented frequency of high titer specific gliadin IgG in ASDs (Table 2) that could be partly explained by the significant increase of AGA-IgG and DPG-IgG (i.e., anti-*α*-gliadin antibodies). Moreover, the fact that AGA-IgA and DPG-IgA titres were similar in ASDs and controls indicates that mucosal surface immune response was possibly not involved [31]. 

Whether or not IgE levels have clinical significance in ASDs is still a debated question [7, 15]. We found no significant differences in total and specific IgE antibody titres in ASDs children versus healthy controls; this could indicate, according to previous studies [16, 17], that these antibodies do not have a valuable clinical significance [15]. The investigated specific IgE also resulted similar among ASDs and controls. In our study it is also shown that the GF/CF diet regimen, as expected, did not influence total or specific IgE titres. 

 Food allergy is related to IgE sensitization [17], and nonallergic food hypersensitivity—such as gluten-sensitivity—could have a role in the gastrointestinal symptoms observed in ASDs children [18]. In addition to an imbalanced intestinal immune response in ASDs children with gastrointestinal symptoms [19], an increased intestinal permeability and altered gut microbiota have been demonstrated [8, 32, 33]. Here we failed to demonstrate a direct correlation between altered barrier function and antibodies increase; as a matter of fact high titers of Ig toward gliadin/gluten were equally distributed in ASDs having normal/impaired intestinal permeability (Figure 3). This lack of correlation could be significant, indicating that different pathways of intestinal damage can be found in ASDs patients, as already suggested [8], given the phenotype heterogeneity of persons with ASDs [7]. 

Although the nature of intestinal damage in ASDs is not yet clearly defined, the inflammatory innate immune responses present in ASDs could predispose children to sensitization to common dietary proteins, leading to GI inflammation and aggravation of some behavioural symptoms [20]. Various etiopathogenetic hypothesis were proposed: mucosal and/or systemic inflammation [19, 34], altered signalling to the tight junctions [35], serum neurotensin increase [36], mast cells intervention [37], molecular mimicry between peptides of dietary gliadin, and a cerebellar peptide, for example, brain protein of Purkinje cells [20, 21]. Sensitivity related illness (SRI) [23] could affect some autistic children; the sensitivity is secondary to a toxicant induced loss of tolerance (TILT) where the primary toxic agents are unknown. Hypersensitivity to gluten and casein, as well as toward other potential antigens, is an acquired condition resulting from a bioaccumulation of toxicants. The GI dysregulation could be the consequence of an abnormal immune response induced by toxicant exposure. Diminishing the toxicant burden (i.e., GF/CF diet) would improve the reactions being, even if only partially, useful.

In this study we have confirmed previous reports [21, 38, 39] suggesting that the prevalence of CD is not increased among children affected by ASD. It is therefore conceivable that gluten could be responsible for causing immune-mediated gastrointestinal symptoms also in ASDs patients without CD, although the exact mechanisms through which this immune response is elicited are still unclear [40, 41]. In CD, the percentage of patients carrying the HLA-DQ2/DQ8 haplotype is approximately 97%, and this haplotype is required to develop this gluten-induced autoimmune enteropathy. In gluten-sensitive individuals these haplotypes are present in about 50% of patients with this condition [42]. In our study, 41% of enrolled ASD patients were HLA DQ2/DQ8 positive, a percentage similar to that found in the normal population. This also supports the hypothesis that a subgroup of ASDs children could be SIR-triggered by gluten. The observation that subjects affected by gluten sensitivity often experience behavioural symptoms [42] further supports this hypothesis.

## 5. Conclusions

 Combined, our data support the hypothesis that immune system of a subgroup of ASDs is triggered by gluten and casein; ASD demonstrates great phenotypic variation, and it could well be related either to AGA and DPG elevated titers or to impaired intestinal barrier function. The determination of antibodies titers to food antigens could be useful to identify the ASDs subjects in whom the implementation of a GF/CF diet might be considered as medical nutrition therapy acting through the elimination of triggering stimuli in an underling SRI condition. Additional investigations are required in order to identify phenotypes based on best and nonresponders to dietary modifications.

## Supplementary Material

Supplementary material includes a table reporting normal values age-based of the investigated total and specific IgE, total IgA and IgG.

## Figures and Tables

**Table 1:** This table reports the % distribution of the investigated ASDs based on the HLA haplotype: 41% bear either one or two alleles.

	HLA: patients			
	Negative	HLA-DQ2	HLA-DQ8	HLA-DQ2/-DQ8
Relative%	59%	33.6%	5.7%	1.6%

**Table 2:** This table is a summary of the results; it reports about all the investigated parameters in all subjects. Number of altered values and % are reported for each investigated parameter.

	ASD: number (%)		Controls: number (%)
Recruited	162		44
Positive for celiac disease (tTg > 9 U/mL, EMA positive)	2		0
Enrolled	160 (M 131; F 31)		44 (M 27; F 17)
HLA DQ2/DQ8 (for details see Table 1)	66 (41.0)		Not determined
Total IgA altered (see age based normal range in Supplementary Material available online at http://dx.doi.org/10.1155/2013/729349)	Deficit: 2 (1.3)		Deficit: 0
	Increase: 13 (8.1)		Increase: 3 (6.8)
Total IgG altered (see age based normal range in Supplementary Material)	0		0
Diet regimen: RD: regular diet = no diet restrictions GF/CF: gluten/casein free diet	RD (*N* = 129)	GF/CF (*N* = 31)	Controls RD (*N* = 44)
LA/MA altered, >0.030	33 (25.6)	8 (25.8)	1 (2.3)
AGA IgA altered, >15 U/mL	3 (2.3)	0	0
AGA IgG altered, >50 U/mL	33 (25.6)	3 (9.7)	4 (9.1)
DPG IgA altered, >5.5 U/mL	3 (2.3)	0	0
DPG IgG altered, >10 U/mL	12 (9.3)	2 (6.5)	0
Total IgE altered (see age based normal range in Supplementary Material)	20 (15.5)	7 (22.6)	4 (9.1)
*β*-Lactoglobulin IgE > 0.35 kUA/L	12 (9.3)	1 (3.2)	2 (4.6)
*α*-Lactalbumin IgE > 0.35 kUA/L	16 (12.4)	3 (9.7)	1 (2.3)
Gluten IgE > 0.35 kUA/L	8 (6.2)	0	2 (4.6)
Casein IgE > 0.35 kUA/L	8 (6.2)	0	0
Milk IgE > 0.35 kUA/L	23 (17.8)	3 (9.7)	2 (4.6)
*β*-Lactoglobulin IgG > 12 mgA/L	30 (23.3)	4 (12.9)	11 (25.0)
*α*-Lactalbumin IgG > 12 mgA/L	27 (20.9)	3 (9.7)	11 (25.0)
Total gliadin IgG (*α*, *β*, *γ*, *ω*) > 12 mgA/L	48 (37.2)	7 (22.6)	10 (22.7)
Casein IgG > 12 mgA/L	45 (34.9)	5 (16.1)	19 (43.2)
