# Supplementary material for: Antibodies against Food Antigens in Patients with Autistic Spectrum Disorders
Source: Biomed Res Int. 2013 Aug 1;2013:729349. doi: 10.1155/2013/729349 (PMC3747333; doi:10.1155/2013/729349)
Supplement: Supplementary file 1 — Supplementary material includes a table reporting normal values age-based of the investigated total and specific IgE, total IgA and IgG. [file 729349.f1.pdf]

Supplemental Material

Table. Normal range cut off values by age, reported for the investigated IgE, IgA and IgG.

**Total IgE normal range: cut off by age**	
0 – 2 years	<40 KU/l
3 - 5	<80
6 – 13	<100
14 - 20	<120
21 – 40	<260
**Specific IgE: Range based evaluation**	
< 0.10 KUA/l	Normal values
0.10 - 0.35	Low levels of IgE to be correlated with clinical aspects
0.35 - 0.7	Low
0.7 - 3.5	Moderate
3.5 - 17.5	High >
17.5 - 100	High >>
> 100	High >>>
**Total IgA normal range: cut off by age**	
1 - 3 years	20-100 mg/dl
4 - 6	27-195
7 - 9	34-305
10 - 11	53-204
12 - 13	58-358
14 - 15	47-249
**Total IgG normal range: cut off by age**	
1 - 3 years	453-916 mg/dl
4 - 6	504-1495
7 - 9	572-1474
10 - 11	698-1560
